# Supplementary material for: Underwater caustics disrupt prey detection by a reef fish
Source: Proc Biol Sci. 2020 Apr 1;287(1924):20192453. doi: 10.1098/rspb.2019.2453 (PMC7209061; doi:10.1098/rspb.2019.2453)
Supplement: Supplementary Figures [file rspb20192453supp1.docx]

Title: Underwater caustics disrupt prey detection by a reef fish

**Authors:**

Matchette, S. R. ^1, 2^ *; Cuthill, I. C. ^1^; Cheney, K. L. ^3, 4^; Marshall, N. J. ^3^; Scott-Samuel, N. E. ^2^

**Affiliations:**

^1^ School of Biological Sciences, Tyndall Avenue, Bristol, BS8 1TQ, UK

^2^ School of Psychological Science, Woodland Road, Bristol, BS8 1TN, UK

^3^ Queensland Brain Institute, University of Queensland, Brisbane, 4072, Queensland, Australia

^4^ School of Biological Sciences, University of Queensland, Brisbane, 4072, Queensland, Australia

* Corresponding author

**Journal:** Proceedings of the Royal Society B: Biological Sciences

**DOI:** 10.1098/rspb.2019.2453

**Supplementary Material**


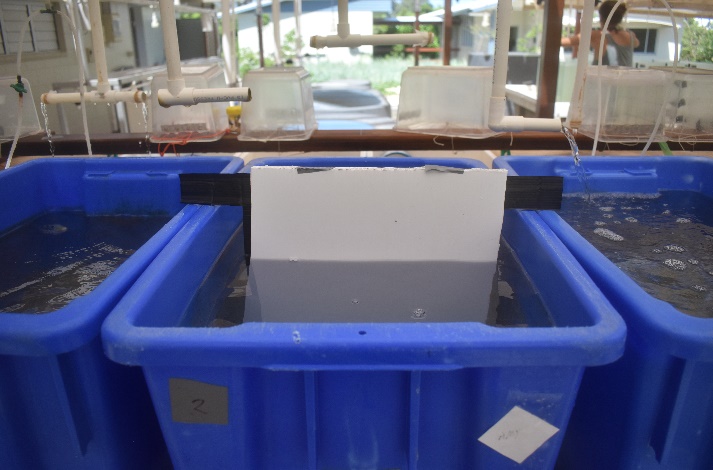


Vertical iPad

25 cm

Shelter

Outlet

Inlet

Camera

Trial divider

40 cm

40 cm

**Figure S1.** A photograph (above; © S. Matchette) and diagram (below) of the individual aquaria. The trial divider was initially inserted to isolate the fish in the left-hand side (or near side in the photo) of the aquaria. The iPad was then lowered into the opposite side and the stimuli video file was loaded. When the iPad was ready, the camera was attached and recording started. The trial divider was then lifted (denoting the start of the trial) and the fish moved from the left side to the right side, to search for and peck on the prey item presented on the iPad (denoting the end of the trial). The shelter was necessary for well-being of the fish throughout the duration of the study and remained in the left-hand side of the aquaria during experimental trials. The water inlet tube, suspended above the right-hand side, was switched off prior to any training or experimental trial.

**
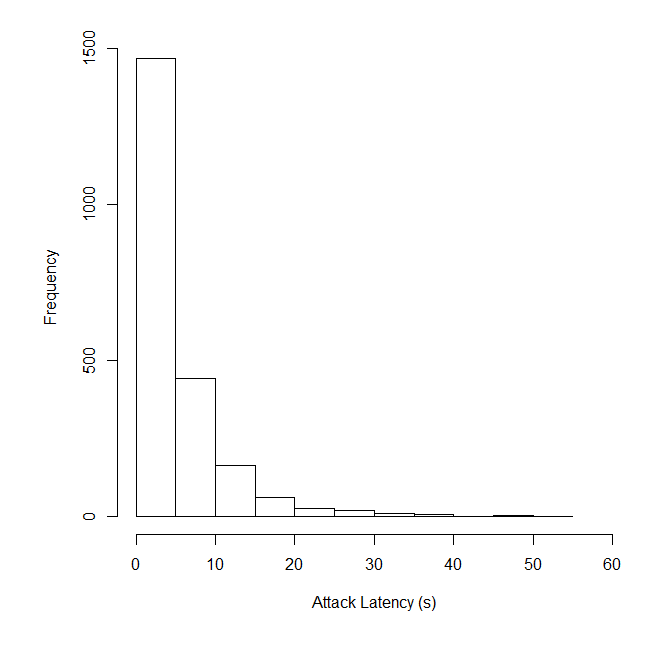
Figure S2.** The frequency of attack latencies across the designated trial time (60 s). Throughout the experimental phase, fish pecked the prey item within a range of 0.6 and 52 s after presenting the stimulus, and within 60 s for 99% of trials (median Attack Latency 3.2 s, interquartile range 4.4 s).

**
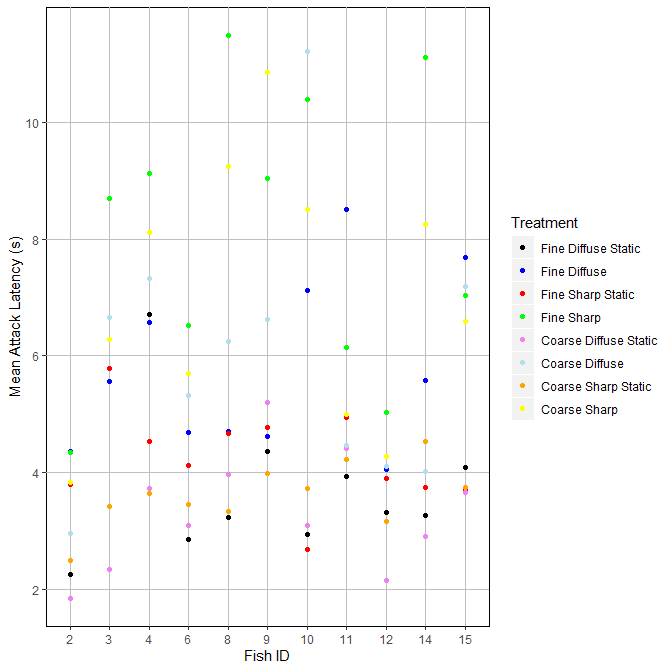
Figure S3.** Mean attack latencies (s) for each triggerfish across eight experimental treatments. The numbering that comprises Fish ID is a product of only 11 fish of the original cohort of 16 fulfilling the training criteria.

**Movie S1.** Picasso triggerfish (*Rhinecanthus aculeatus*) exhibiting escape behaviour within their shallow reef flat habitats off Casuarina Beach, Lizard Island, Great Barrier Reef, Australia (14°40 8 S, 145°27 34 E). Fish were filmed at 0.5 m under unsettled wave conditions with an AKASO V50 Pro (Akaso, [www.akaso.net](http://www.akaso.net); 4K resolution, 30 fps and 170° viewing angle). Movie © S. Matchette.

**Movie S2 and S3.** Example trial clips for fish searching for moving prey items within fine scale diffuse water caustics, both dynamic (video 2) and static (video 3). The removal of the divider started the given trial, while a peck on the prey item signified the end of the trial; upon a peck, a food reward is provided and the divider refitted. Movies © S. Matchette.
